# QSKL protects against myocardial apoptosis on heart failure via PI3K/Akt-p53 signaling pathway

**DOI:** 10.1038/s41598-017-17163-x

**Published:** 2017-12-05

**Authors:** Hong Chang, Chun Li, Qiyan Wang, Linghui Lu, Qian Zhang, Yi Zhang, Na Zhang, Yong Wang, Wei Wang

**Affiliations:** 1 School of Life Sciences, Beijing University of Chinese Medicine, Bei San Huan Dong Lu 11, ChaoYang District, Beijing, 100029 China; 2 Traditional Chinese Medicine College, North China University of Science and Technology, No. 21 Bohai Road, Caofeidian New City, Tangshan, Hebei, 063210 China; 3 Modern Research Center for Traditional Chinese Medicine, Beijing University of Chinese Medicine, Bei San Huan Dong Lu 11, ChaoYang District, Beijing, 100029 China; 4 School of Chinese Materia Medica, Beijing University of Chinese Medicine, Bei San Huan Dong Lu 11, ChaoYang District, Beijing, 100029 China; 5 Beijing University of Chinese Medicine, Bei San Huan Dong Lu 11, ChaoYang District, Beijing, 100029 China

## Abstract

The ancient traditional Chinese medicine Qishenkeli (QSKL) is widely used in the treatment of heart failure (HF) in China. Previous studies have shown that QSKL has definite effects on HF. The purpose of this study is to identify the regulation of QSKL on apoptosis and clarify the underlying mechanism. An apoptosis model of H9C2 cells was induced by oxygen-glucose deprivation/recovery (OGD/R). An animal model of HF was induced by ligation of left anterior descending (LAD) coronary artery in rat. We found that QSKL reduced intracellular ROS generation, increased mitochondrial membrane potential and protected H9C2 cells against OGD/R-induced apoptosis. *In vivo* results showed that QSKL administration could improve cardiac functions, decrease fibrotic area, infarct size and apoptotic rate in HF model. QSKL regulated the expressions of key apoptotic molecules, including increasing Bcl-2/Bax ratio, reducing the expressions of P53, Bax and Cleaved-caspase-3. Interestingly, QSKL also regulated the phosphorylated expressions of PI3K and Akt without significantly affecting PTEN. Taken together, the protective and anti-apoptotic effects of QSKL could be mediated partly through modulating the PI3K/Akt-P53 apoptotic pathway.

## Introduction

Heart failure (HF), a terminal stage of most cardiovascular diseases, has been recognized as a refractory condition worldwide. In China, there are over 4 million patients with HF and the mortality rate is much higher than that in developed countries^1^. Investigation into the mechanisms of HF has been an ongoing effort and apoptosis has been shown to contribute to the progress of HF. Cardiomyocytes apoptosis occurs in the development of various cardiovascular diseases, such as myocardial infarction and ischemia/reperfusion^2,3^. In addition, apoptotic cells around infarcted tissue have been reported to play an important role in the progression of cardiac remodeling^4^. It has been proven that inhibition of cellular apoptosis could significantly improve left ventricular remodeling and chronic heart failure^5^. In particular, P53-Caspase-3 mediated apoptotic signaling pathway is considered to be critical during HF^6^.

Regulation of cell apoptosis is one of the roles that P53 plays in a variety of diseases. P53 could not only up-regulate Bax expression, but also inhibit the anti-apoptotic effect of Bcl-2 by directly combining with it^7^. Moreover, elevated P53 expression induced by increased intracellular ROS could also lead to cell apoptosis^8^. Studies have found that dysregulation of P53 is related to the progression of various cardiovascular diseases, such as ischemia/reperfusion, heart failure, atherosclerosis, etc^9–11^. Therefore, inhibition of apoptosis through down-regulating the activity of P53 protein will provide theoretical basis for the prevention and treatment of HF.

Qishenkeli (QSKL), one of traditional Chinese medicine formulae, is an effective compound in the treatment of HF. Previous studies showed that the mechanisms of QSKL in preventing HF may be mediated through attenuating oxidative stress, inhibiting inflammation and regulating renin-angiotensin-aldosterone system (RAAS)^12–14^. The anti-apoptotic effect of QSKL hasn’t been illuminated yet. In this study, a heart failure (HF) rat model and an apoptotic H9C2 cell model were induced to investigate the underlying mechanisms of QSKL on inhibiting myocardial apoptosis.

## Results

### QSKL restored cardiac functions

**Figure 1:**
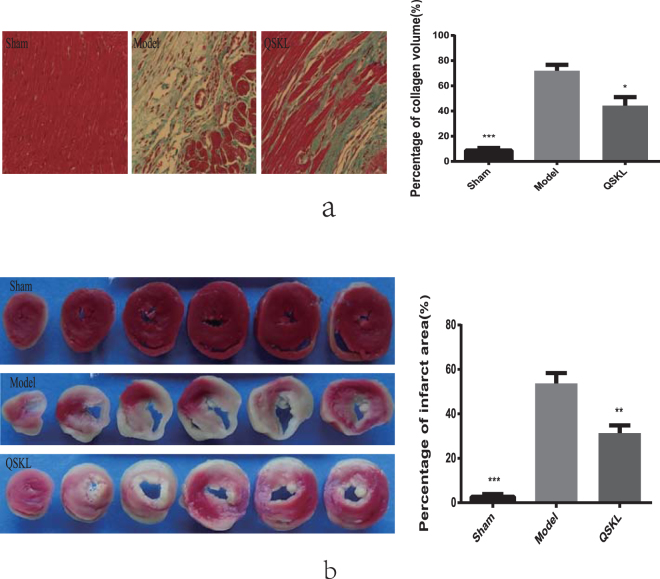
Effects of QSKL on Masson staining and infarct size. (**a**) Masson staining in different groups. Masson staining revealed different degree fibrosis in different groups. In sham group, there was almost no fibrotic area. While in model group, there was significant fibrotic area. The increased fibrotic area was significantly attenuated in QSKL group by contrast with this in model group. Magnification: 200×. (**b**) Heart cross-sections and infarct size. Each panel showed 6 heart cross-sections of one rat in each group. Infarct size was calculated as the percentage of area to the left ventricular cross-section. Data were expressed as mean ± SD. *P < 0.05, **P < 0.01, ***P < 0.001 versus model.

28 days after surgery, echocardiography was used to assess cardiac functions. Compared with sham group, LVEDd and LVEDs in model group increased significantly, indicating the enlargement of cardiac chamber. Meanwhile, values of EF and FS dropped sharply, suggesting serious impairment of heart function. In QSKL group, LVEDd and LVEDs were down-regulated and values of EF and FS were up-regulated, indicating that QSKL could improve heart function (Table 1). Masson trichrome staining was performed to assess myocardial fibrosis. Compared with sham group, thickening and lengthening of myocardial fibers could be observed in model group (8.67 ± 3.51% vs 72 ± 8.19%), suggesting severe fibrosis in local tissue. QSKL significantly attenuated the fibrosis degree (44.33 ± 11.68% vs 72 ± 8.19%) (Fig. 1a). Moreover, myocardial infarct size was measured by staining with 2,3,5-triphenyltetrazoliumchloride (TTC). As shown in Fig. 1b, little infarction was observed in sham group (2.67 ± 2.08%) and the infarct area increased in model group (53.67 ± 8.14%). QSKL could decrease the infarct area in comparison to model group (31.33 ± 6.03%).

**Table 1:** The cardiac functions detected by echocardiography in different groups of rats.

Group	LVEDd/cm	LVEDs/cm	FS%	EF%
Sham	0.58 ± 0.074**	0.32 ± 0.048**	44.62 ± 5.163**	81.05 ± 4.907**
Model	0.87 ± 0.035	0.67 ± 0.044	22.57 ± 2.229	50.85 ± 4.272
QSKL	0.69 ± 0.077**	0.45 ± 0.101**	35.22 ± 7.137**	69.78 ± 10.459**

Data were expressed as mean ± standard deviation (SD) (n = 6). **P < 0.01 versus model.

### Effects of on N-terminal pronatriuretic peptide (NT-proBNP), lactate dehydrogenase (LDH), creatine kinase (CK), superoxide dismutase (SOD) and malondialdehyde (MDA)

Bio-markers of heart failure including NT-proBNP, LDH and CK were detected to evaluate the effect of QSKL^15,16^. In model group, levels of NT-proBNP, LDH and CK were increased compared with sham group. Treatment with QSKL resulted in decreased release of NT-proBNP, LDH and CK as compared with model group. Oxidative stress plays critical role in the progression of HF and SOD is an important anti-oxidative enzyme^17^. In model group, SOD activity decreased and MDA increased, suggesting the existence of oxidative stress in HF model. Treatment with QSKL could up-regulate SOD activity and depress MDA level, indicating that QSKL has anti-oxidative effect (Table 2).

**Table 2:** Effects of QSKL on the biochemical parameters in serum of rats.

Group	NT-proBNP(pg/mL)	LDH(U/L)	CK(U/L)	SOD(U/mL)	MDA(nmol/mL)
Sham	15.41 ± 6.11**	301.83 ± 64.067**	365.5 ± 62.513*	145.78 ± 7.438**	3.94 ± 0.38*
Model	42.79 ± 3.58	482.67 ± 62.369	491.17 ± 52.943	128.38 ± 8.797	4.78 ± 0.689
QSKL	27.46 ± 2.04**	349.67 ± 17.58**	373.2 ± 19.652**	140.43 ± 4.682*	3.87 ± 0.377**

Data were expressed as mean ± SD (n = 6). ^*^ *P* < 0.05, ^**^ *P* < 0.01 versus model.

### Effect of QSKL on apoptosis and apoptosis-related proteins

**Figure 2:**
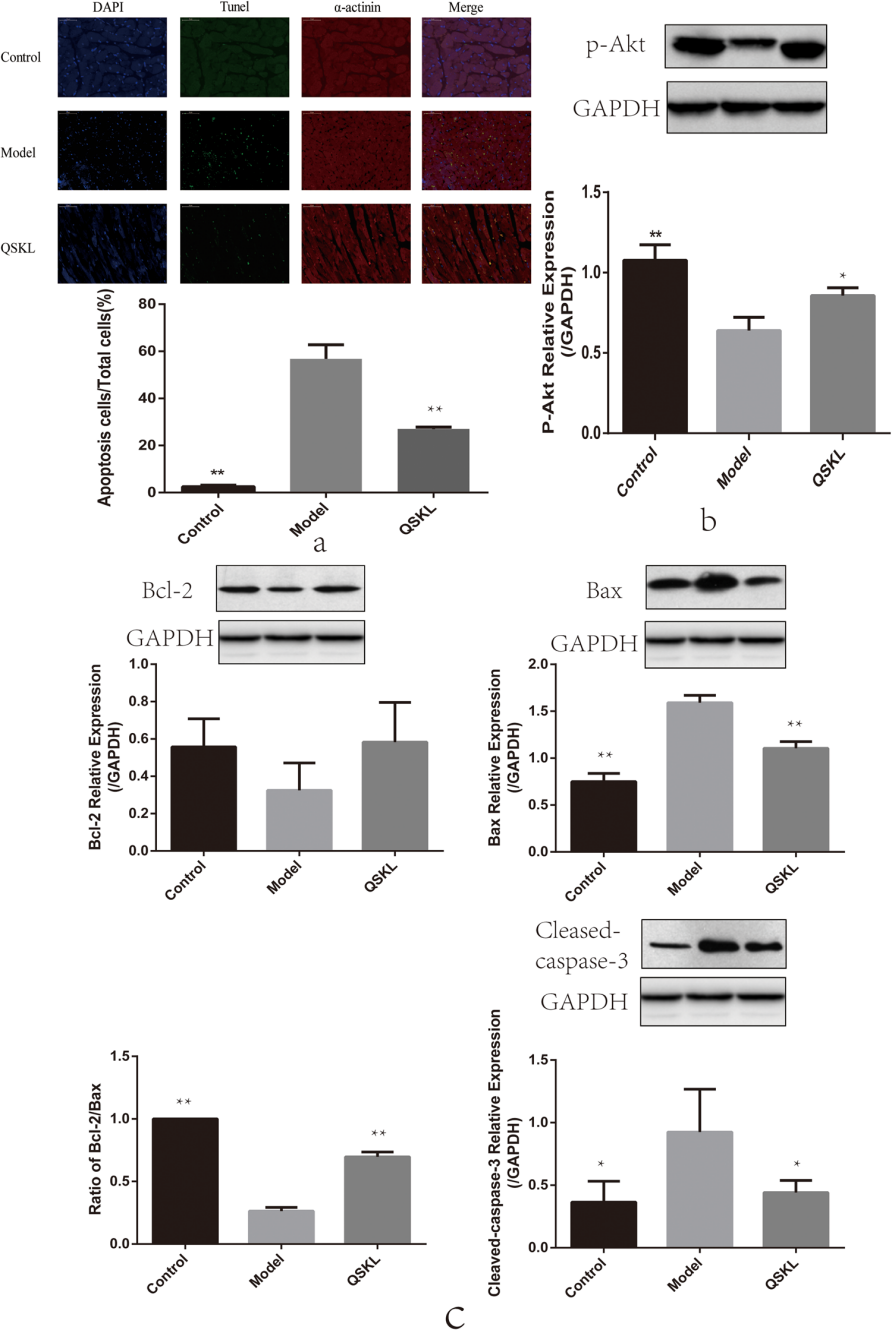
Effects of QSKL on apoptosis and apoptosis-related proteins in heart tissues of HF model of rats. (**a**) Apoptotic rate in infarct border zone of heart tissue was higher in model group compared with control group and QSKL treatment reduced apoptotic rate. (**b**) QSKL modulated p-Akt expression. (**c**) QSKL modulated expressions of apoptosis-related proteins, including Bcl-2, Bax and Cleaved-caspase-3. Cells were examined by a fluorescence microscope (200×). The expression of GAPDH was measured as an internal control. Data were expressed as mean ± SD. *P < 0.05, **P < 0.01 versus model.

As shown in Fig. 2a, apoptotic cells number in infarct border zone of heart tissue was higher in model group and treatment of QSKL reduced apoptotic cells number. Furthermore, expressions of key molecules in the apoptotic signaling pathway including Bax, Bcl2 and Cleaved-caspase-3 were measured by western blot. As shown in Fig. 2c, the expressions of Bax and Cleaved-caspase-3 increased, whereas the expressions of Bcl-2 decreased in model group compared to control group, indicating that the apoptotic signaling pathway was activated. Treatment with QSKL could restore the expressions of these proteins towards normal levels, suggesting a protective effect against apoptosis.

### Effect of QSKL on OGD/R-induced loss of cell viability

**Figure 3:**
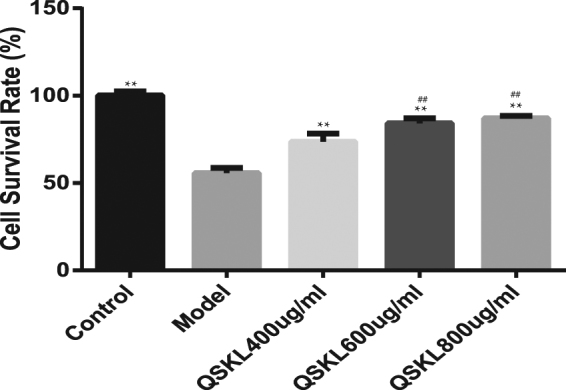
QSKL rescued OGD/R-induced loss of cell viability. H9C2 cells were incubated with different concentrations of QSKL (400, 600, 800 μg/ml) during the process of OGD/R. Cellular viability was detected with MTT assay. Data were expressed as mean ± SD. ^**^ *P* < 0.01 versus model; ^##^ *P* < 0.01 versus 400 μg/ml QSKL.

The effects of QSKL on OGD/R-induced cytotoxicity were detected with MTT assay. As shown in Fig. 3, treatment of QSKL (400, 600, 800 μg/ml) provided significant protective effects against OGD/R-mediated cytotoxicity (*P* < 0.01). Cells treated with 600 μg/ml QSKL had significantly higher viability than those treated with 400 μg/ml QSKL (*P* < 0.01), whereas cells treated with 600 μg/ml and 800 μg/ml QSKL had similar viabilities. Therefore, 600 μg/ml QSKL was applied to treat cells to assess the effects of QSKL in the subsequent experiments. Furthermore, LY294002, an inhibitor of PI3K/Akt pathway, was applied to normal H9C2 cells to assess its effect on cell viability. The nontoxic concentration range of LY294002 was tested and result showed that 20 μM was the suitable nontoxic concentration for the subsequent experiments (Supplement 1).

### QSKL attenuated intracellular ROS generation

**Figure 4:**
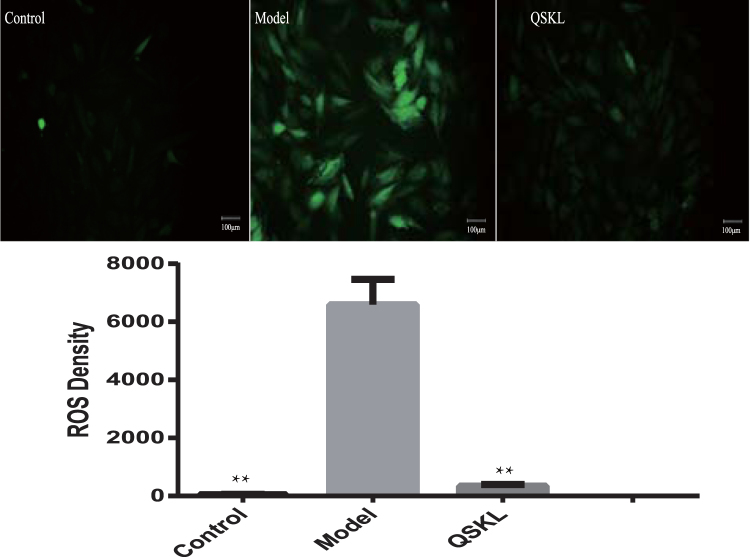
Intracellular ROS induced by OGD/R with/without QSKL. H9C2 cells were incubated with/without QSKL during the process of OGD/R. ROS were detected with DCFH-DA assay. QSKL down-regulated production of ROS in the process of OGD/R. Fluorescent intensity was examined under a fluorescence microscope. Image J was used to analyze the data. Magnification: 100×. Data were expressed as mean ± SD. ^**^ *P* < 0.01 versus model.

ROS induced by OGD/R could promote cellular damage. To determine whether treatment with QSKL could alleviate OGD/R-induced oxidative stress, the treated cells were measured with DCFH-DA assay. Fluorescent intensity was examined under a fluorescence microscope. As shown in Fig. 4, intracellular ROS significantly increased in OGD/R-treated H9C2 cells compared with that in the control group. After treatment with QSKL, intracellular ROS was significantly reduced compared with that in OGD/R-treated cells.

### QSKL inhibited cellular apoptosis

**Figure 5:**
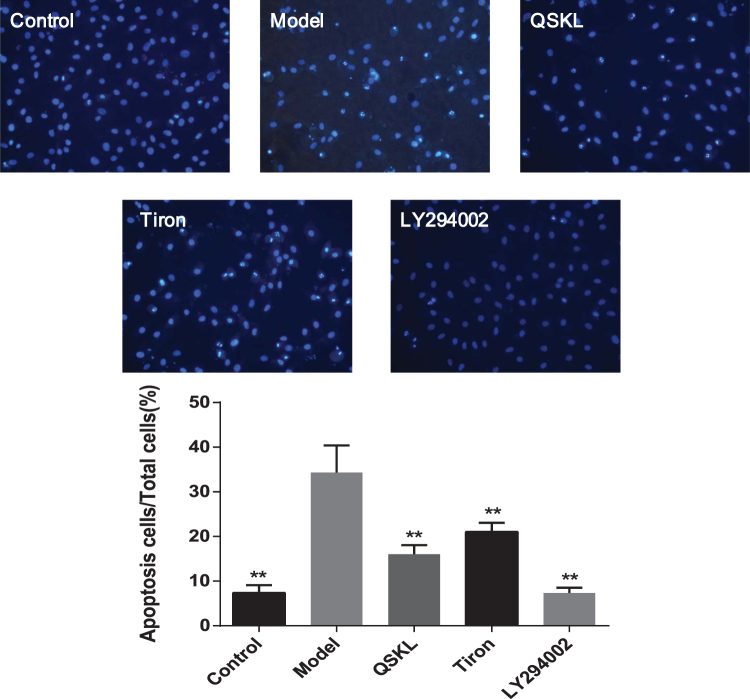
Cell apoptosis induced by OGD/R with/without QSKL. H9C2 cells were incubated with/without QSKL during the process of OGD/R. Cellular apoptosis was detected with Hoechst 33342 assay. Apoptotic rates in control, model, QSKL, Tiron and LY294002 groups were shown. Apoptotic rate in model group increased, while QSKL and Tiron had a protective effect against apoptosis. Apoptotic rate in LY294002 group was similar as that in control group. Fluorescent intensity was examined under a fluorescence microscope. Magnification: 200×. Data were expressed as mean ± SD. ^**^ *P* < 0.01 versus model.

Hoechst 33342 assay was used to detect cellular apoptosis in H9C2 cells. Distinct morphological nuclear changes, such as chromatin shrinking, the occurrence of highlight dying, chromatin condensation and fragmentation, were observed under fluorescent microscopy. As shown in Fig. 5, apoptotic rate was much higher in model group than in control group (*P* < 0.01). Treatment with QSKL down-regulated apoptotic rate significantly, suggesting that it possesses anti-apoptotic property. Apoptotic rate in LY294002 group was similar as that in control group. To determine if apoptosis was induced by the ROS, disodium 4,5-dihydroxy-1,3-benzenedisulfonate (tiron), a cell permeable free radical scavenger, was applied to model cells. The results showed that apoptotic rate in tiron group was remarkably down-regulated as compared with model group, demonstrating that ROS played a critical role in inducing apoptosis.

### QSKL attenuated OGD/R-induced mitochondrial membrane potential loss in H9C2 cells

**Figure 6:**
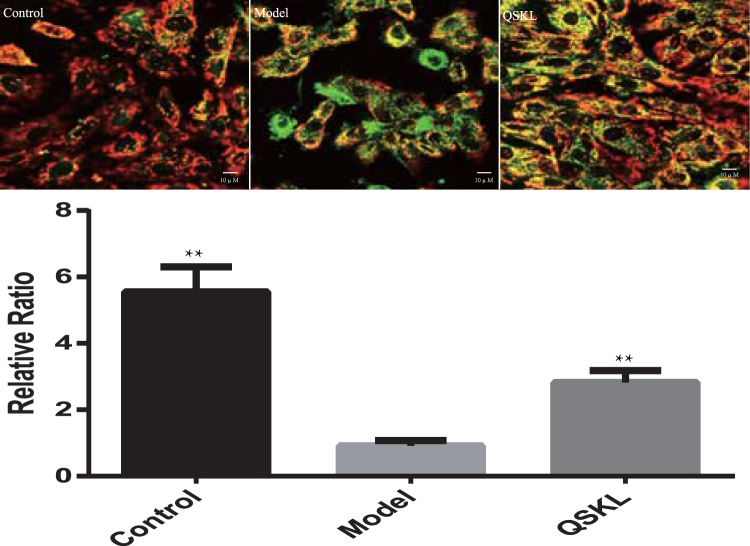
Changes of mitochondrial membrane potential (ΔΨm) levels induced by OGD/R with/without QSKL. H9C2 cells were incubated with/without QSKL during the process of OGD/R. Mitochondrial membrane potential was assessed with JC-1 assay. Fluorescent intensity was examined under a fluorescence microscope. Treatment with QSKL up-regulated mitochondrial membrane potential in H9C2 cells. Magnification: 600×. Composite red and green fluorescence were shown. Data were expressed as mean ± SD. ^**^ *P* < 0.01 versus model.

The mitochondrial membrane potential is an essential factor in maintaining the integrity of mitochondria and regulating cell apoptosis^18^. Loss of mitochondrial membrane potential is an early manifestation of apoptosis. Composite red and green fluorescence is shown in Fig. 6. In control group, stained mitochondria emitted red fluorescence. Cells treated with OGD/R exhibited more green fluorescence in the cytoplasm and less red fluorescence in the mitochondria, suggesting loss of mitochondrial membrane potential. Treatment of QSKL could enhance the red fluorescence and attenuate the loss of mitochondrial membrane potential induced by OGD/R.

### Effects of QSKL on the expressions of apoptosis-related proteins

**Figure 7:**
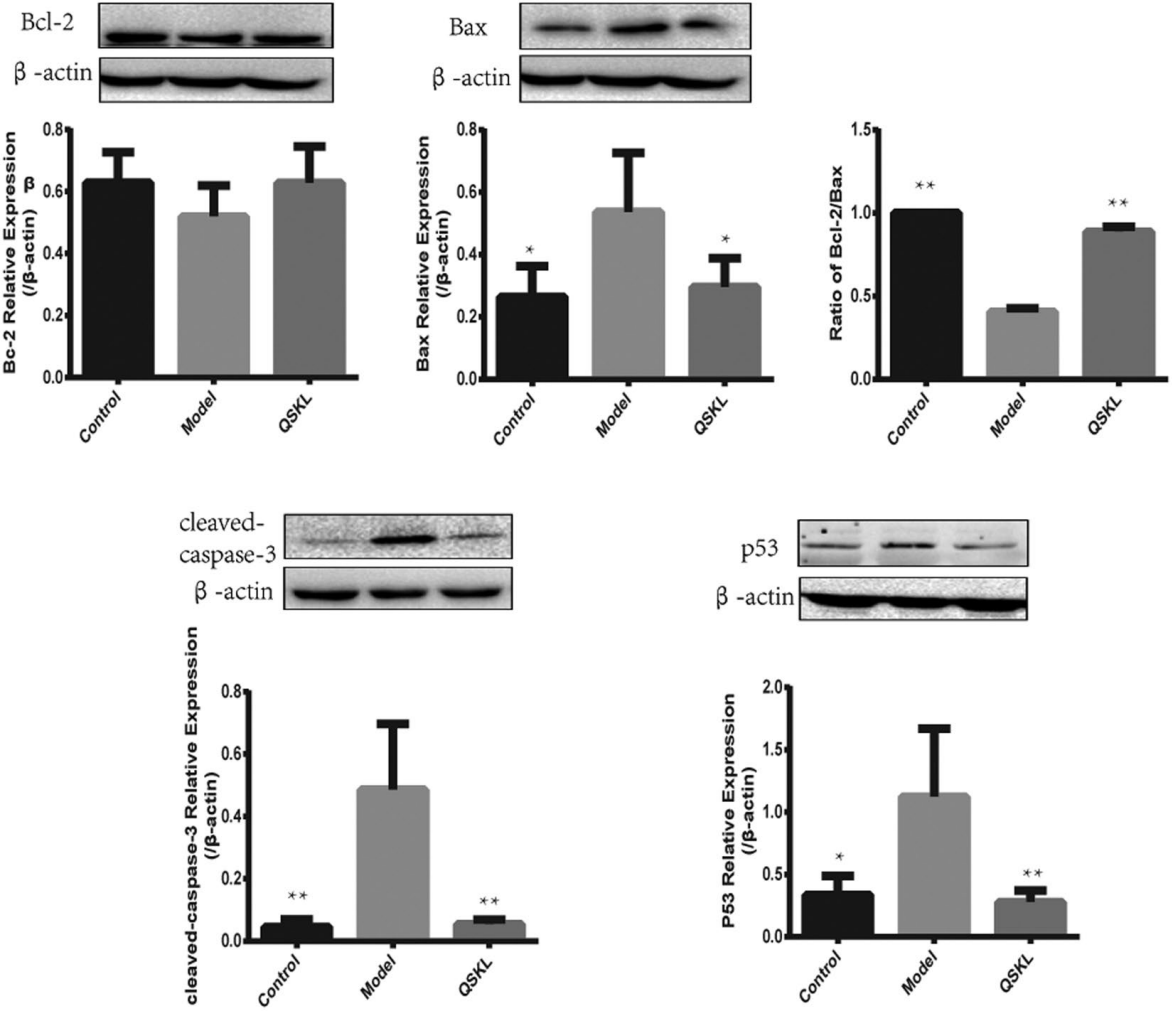
QSKL modulated expressions of apoptosis-related proteins in H9C2 cells. H9C2 cells were incubated with/without QSKL during the process of OGD/R. QSKL regulated expressions of Bcl-2, Bax, Cleaved-caspase-3 and P53 in H9C2 cells. The expression of β-actin was measured as an internal control. Data were expressed as mean ± SD. *P < 0.05, **P < 0.01 versus model.

We further investigated the mechanism of QSKL in protecting cells against apoptosis by *in vitro* studies. As shown in Fig. 7, the expression of Bcl-2 and the ratio of Bcl-2/Bax decreased, whereas the expressions of Bax, Cleaved-caspase-3 and P53 increased in H9C2 model group compared to control group, indicating that the apoptotic signaling pathway was activated by the process of OGD/R. Expressions of these proteins reverted toward normal levels in treatment of QSKL. The results showed that the protection of QSKL against cell apoptosis may be mediated through P53-Caspase-3 apoptotic signaling pathway.

### Effect of QSKL on PI3K/Akt pathway

**Figure 8:**
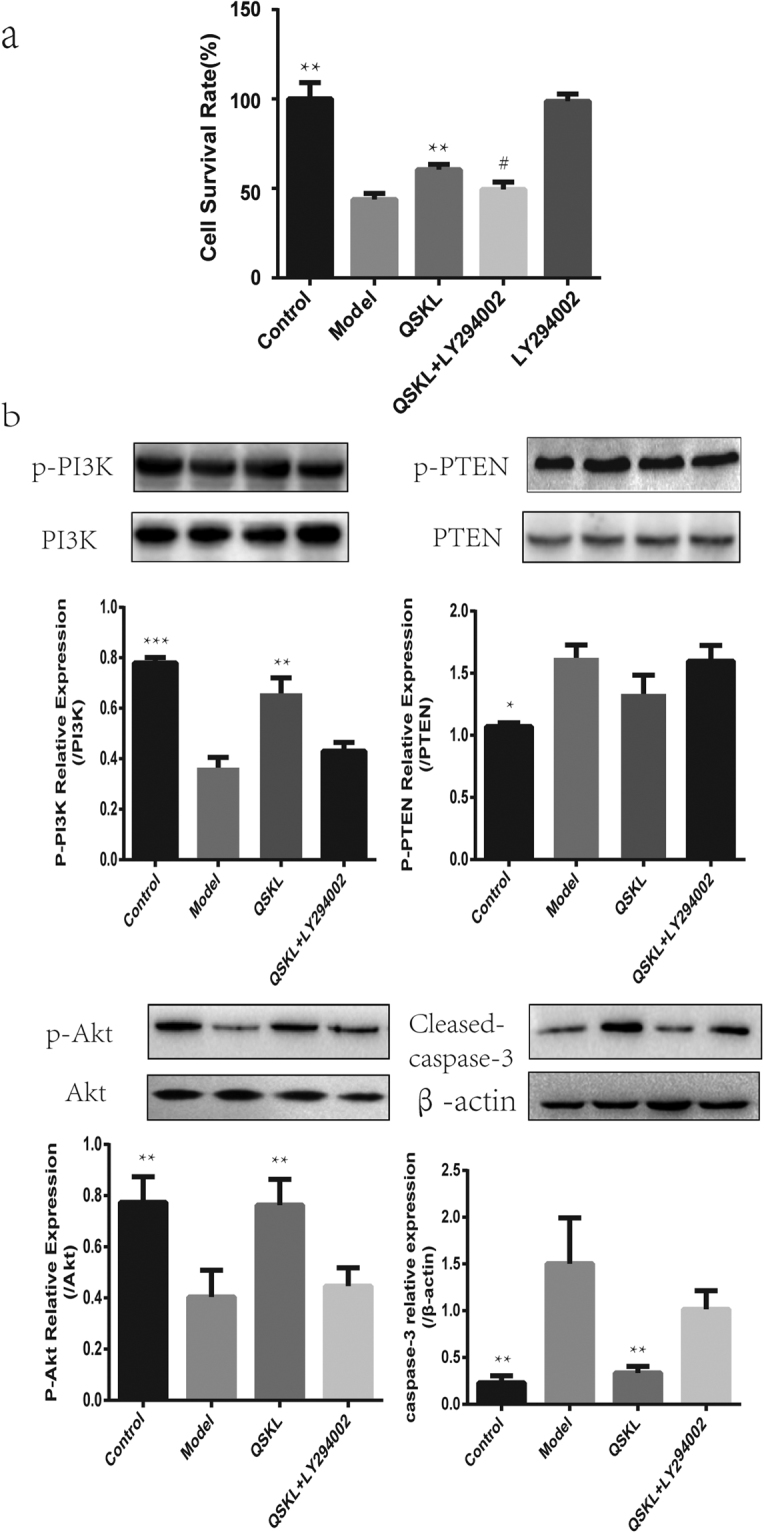
Effect of QSKL on PI3K/Akt pathway. (**a**) Effects of QSKL and LY294002 on H9C2 cell survival rate. QSKL protected against OGD/R-induced cell loss. LY294002 abolished the protective effect of QSKL. (**b**) The effects of QSKL on p-PI3K and p-PTEN and inhibition of p-Akt abolished the effect of QSKL on apoptotic proteins. QSKL up-regulated p-PI3K and p-Akt and down-regulated the expression of Cleaved-caspase-3, but had no significant effect on p-PTEN expression. Co-incubation of cells with LY294002 abolished the regulative effects of QSKL. The expression of β-actin was measured as an internal control. Data were expressed as mean ± SD. *P < 0.05, **P < 0.01, ***P < 0.001 versus model; ^#^ *P* < 0.05 versus QSKL.

The effects of QSKL on Akt signaling pathway were investigated by both *in vitro* and *in vivo* studies. Cell viability decreased dramatically in H9C2 model group, while treatment of QSKL protected against QGD/R-induced loss of cell viability. After addition of LY294002, an inhibitor of PI3K/Akt pathway, the protective effect of QSKL was attenuated (Fig. 8a). Expressions of PI3K/Akt pathway related proteins were further detected by western blot. Expressions of both p-PI3K and p-Akt were decreased while p-PTEN was increased in model group compared with control group (Fig. 8b). QSKL could up-regulate the expressions of p-PI3K and p-Akt, down-regulated the expression of Cleaved-caspase-3, but p-PTEN expression didn’t show statistic difference. However, LY294002 abolished the regulative effects of QSKL (Fig. 8b). Moreover, QSKL could up-regulate p-Akt expression in HF model of rats (Fig. 2b). These results suggested that QSKL modulated apoptosis at least partly by acting on p-PI3K/p-Akt signaling pathway.

## Discussion

Apoptosis has been shown to play an important role in the progression of heart failure. In this study, we demonstrated that QSKL could exert cardio-protective effect by attenuating cardiac apoptosis. Further *in vitro* and *in vivo* studies suggested that the anti-apoptotic effect of QSKL was potentially exerted by regulating PI3K/Akt-p53 signaling pathway.

It has been reported that the occurrence of HF is not only caused by the reduction of myocardial contractility, but also the increase of apoptotic cell number^19^. Apoptosis plays a critical role in the process of ventricular remodeling and heart failure^4,20^. The Bcl-2 protein family determines the commitment of cells to apoptosis and activation of Caspase-3 triggers the execution of cell apoptosis. In the Bcl-2 protein family, there are both pro-apoptotic proteins and anti-apoptotic proteins. Bax, in the form of oligomers, can prompt the formation of membrane pores and release pro-apoptotic substances into the cytoplasm, exerting pro-apoptotic function, whereas Bcl-2 inhibits cell apoptosis by blocking oligomerization of pro-apoptotic proteins^21^. Besides, P53 is also closely related to the apoptotic pathway. It can not only up-regulate Bax expression, but also inhibit the anti-apoptotic effect of Bcl-2 by directly combining with it^7^. Several stimuli can lead to cellular apoptosis. ROS can induce oxidative stress and is one of the main factors affecting cardiomyocytes apoptosis^22^. A variety of external stimulation can initiate the reactive increase of ROS, thereby inducing oxidative stress. ROS are mainly produced in mitochondria, where electron transport chains are located and cellular respiration happens. Mitochondrial dysfunction has been shown to participate in the induction of apoptosis and loss of mitochondrial membrane potential (MMP) may be an early event in the apoptotic process^23^.

In this study, an animal model of HF and OGD/R-induced apoptosis model of H9C2 cells were established to explore the effects of QSKL on cardiac apoptosis. Consistent with previous studies^12^, animals in model group were characterized by declined diastolic and systolic myocardial performance and increased fibrotic region. Heart functions were impaired in model group. In the treatment group, EF was increased by 18.93%, fibrosis was attenuated, indicating that QSKL possesses cardiac-protective function. Biomarkers of heart failure, such as NT-proBNP, LDH and CK^15,16^, are indicators of myocardial damage and the imbalance between SOD and MDA indicates myocardial oxidative damage^24^. In model group, NT-proBNP, LDH and CK increased remarkably and the balance between SOD and MDA was disrupted. QSKL inhibited release of NT-proBNP, LDH and CK and restored the balance between SOD and MDA, suggesting that QSKL could mitigate myocardial damage. Moreover, we found that QSKL inhibited myocardial apoptosis in the border zones of ischemic heart tissues. To further evaluate the anti-apoptotic efficacy, we explored the effect of QSKL by *in vitro* study. Treatment with QSKL increased cell viability in OGD/R-stimulated cells and attenuated generation of intracellular ROS. Apoptotic rate in treatment with QSKL were much lower than in model group. Furthermore, QSKL treatment up-regulated mitochondrial membrane potential in H9C2 cells. Based on these data, we demonstrated that QSKL has protective effects against OGD/R-induced apoptosis via inhibiting generation of ROS and up-regulating mitochondrial membrane potential.

The mechanism of QSKL on inhibiting cellular apoptosis was further investigated. Expressions of key molecules in the apoptotic pathways, such as Bcl-2, Bax, Cleaved-caspase 3 and P53, were measured by western blot. The balance between Bcl-2 and Bax was disrupted and expression of Cleaved-caspase-3 was increased, suggesting that the apoptotic pathway was activated in cell model and HF model. QSKL treatment could increase Bcl-2 level, suppress Bax expression and reduce Cleaved-caspase-3 expression. Moreover, QSKL suppressed the expression of P53 in H9C2 cells. To further identify the mechanism of QSKL on apoptosis, LY294002, an inhibitor of PI3K, was used to study the effect of QSKL on PI3K/Akt signaling pathway. LY294002 is a protein kinase inhibitor that can block the signal transduction of PI3K^25^. As an important signal transduction pathway, PI3K/Akt plays an important role in cell survival, apoptosis and proliferation^26^. Meanwhile, PTEN, a dual lipid and protein phosphatase, negativly regulates PI3K activity^27,28^. Our study found that the protective effect of QSKL was attenuated when LY294002 was added to cell culture. Expressions of phosphorylated PI3K and Akt were increased in QSKL treatment group and administration of LY294002 reduced p-Akt and p-PI3K expressions. Furthermore, co-administration of LY294002 also increased expression of Cleaved-caspase-3, indicating that the anti-apoptotic effect of QSKL were blocked by LY294002. Expression of p-PTEN was not affected by QSKL treatment. These results suggest that QSKL may exert anti-apoptotic effect by regulating p-PI3K and p-Akt instead of interacting directly with PI3K and Akt. Some of the components in QSKL have been proven to have anti-apoptotic effect. For example, quercetin, luteolin and tanshinone IIA have been shown to have anti-apoptotic effects on myocytes^29–31^. Moreover, we applied network pharmacology to evaluate the effective ingredients and respective targets of QSKL and selected components related to apoptosis. The results showed that six ingredients, including quercetin, kaempferol, luteolin, β-sitosterol, tanshinone IIA and naringenin, have the potential anti-apoptotic effect (Supplement 2). In the future studies, we will validate the effects of these potentially effective ingredients by *in vivo* and *in vitro* experiments.

In conclusion, we investigated the protective effects of QSKL in HF animal model and apoptosis model of H9C2 cells in this study. The results demonstrated that QSKL can prevent cardiomyocytes apoptosis partly through regulating PI3K/Akt-p53 signaling pathway. This study provides basis for further research of effective substance in the treatment of HF.

## Material and Methods

### Materials

Thiazolyl Blue Tetrazolium Bromide (MTT) and 2′,7′-Dichloroflurescin diacetate (DCFH-DA) were obtained from Sigma (USA). Penicillin, streptomycin and 0.05% trypsin were purchased from Invitrogen (USA). Dulbecco’s modified Eagle’s medium (DMEM) was purchased from Hyclone (USA). Fetal bovine serum (FBS) was from Corning (USA) and Tiron was from SigmaeAldrich (USA). A transferasemediated uridine nick end labeling (TUNEL) detection kit was obtained from Roche (Germany). Mitochondrial membrane potential assay kit with JC-1 and Hoechst33342 staining assay were from Beyotime Technology (China). Earle’s balanced salt solution was from Aladdin (China). The primary antibodies against p-Akt, and Cleaved-caspase-3 were from Cell Signaling Technology (USA) and antibodies against P53, Bcl-2 and Bax were from Abcam (USA). Antibodies against β-actin, GAPDH and secondary antibodies were purchased from Beijing Zhong Shan Jinqiao Biological Technology Co., Ltd (China). LY294002 was obtained from Selleck (USA). Freeze-dried powder of Qishenkeli (QSKL) was prepared by College of TCM, Beijing University of Chinese Medicine. QSKL and LY294002 were dissolved in DMSO or DMEM for *in vitro* assay as storage concentration.

### Animals

The experiment was performed in accordance with the Guide for the Care and Use of Laboratory Animals published by the National Institutes of Health and approved by the Animal Care Committee of Beijing University of Chinese Medicine. Adult male Sprague-Dawley (SD) rats (weighting 230–250 g) in SPF grade were purchased from Beijing Vital River Laboratory Animal Technology Co. Ltd (SCXK2010~2011).

### HF model preparation and grouping

Left anterior descending (LAD) coronary artery of rats was ligated to induce HF model as previous study described^12^. Briefly, after anaesthetizing with 1% pentobarbital sodium (50 mg kg^−1^ intraperitoneally), rats underwent left thoracotomy between the third and fourth intercostal space and the heart was exposed. The LAD coronary artery was ligated with a 5–0 polypropylene suture (Shuangjian, Shanghai, China) 1 millimeter below the left atrium. Then the thorax was closed. The rats in sham group were prepared following an identical procedure, but had no actual ligation of LAD artery. Operated rats were divided into two groups: the model group and QSKL group. The third group was the sham group. The rats in QSKL group were treated with a total daily dose of 2.33 g/kg QSKL dissolved in water for 28 days. QSKL has similar components as QSYQ we used in our previous studies but the preparation technologies have been improved^13^. In QSKL formula, Radix Aconiti Lateralis Preparata was boiled for 4 to 6 hours before drying and distribution. Prior to use, Radix Aconiti Lateralis Preparatait was pre-decocted for 30 to 60 minutes again before the addition of other herbs to reduce its potential toxicity^32^. Rats in sham group and model group were not given any treatment except the same volume of water. At the end of the study, all rats were assessed by echocardiography following an overnight fasting. Heart tissue samples were stored in 4% paraformaldehyde for further use. Serum samples were stored at −80 °C until analyzed.

### Echocardiographic assessment of cardiac functions

Echocardiography was used to assess the left ventricular end-systolic diameter (LVEDs), left ventricular end-diastolic diameter (LVEDd), ejection fraction (EF) and fractional shortening (FS). The LV dimension (LVD) was measured using m-model fractional shortening and FS% was calculated using the following equation: FS% = [(LVEDd−LVEDs)/LVEDd] × 100%.

### Morphometric analysis

Myocardial tissues in the left ventricle (LV) of sacrificed rats (approximately 2 mm in thickness) were removed. Samples were fixed in 4% pre-cooled paraformaldehyde for 72 h and embedded in paraffin for histological studies. Paraffin-embedded tissues were sectioned into slices about 5 μm thicknesses. Masson staining was performed to assess myocardial fibrosis. Images were visualized under an optical microscope at ×400 magnification.

### Measurement of infarct size

Myocardial infarct size was measured by staining with TTC. The hearts were sliced into slices of 2 mm thickness, incubated in 1% TTC at 37 °C for 20 minutes, followed by fixation in 4% paraformaldehyde for 10 minutes. Image-J was used to analyze the infarcted area. The results were defined as myocardial infarct size/left ventricular cross-sectional area × 100%.

### Detection of LDH, CK-MB, SOD and MDA

After dissolution, the serum samples were collected by centrifugation at 1000 g for 5 minutes at 4 °C. CK-MB and LDH activities in serum were determined using a Hitachi 17080 Automatic Biochemical Analyzer (Hitachi Co., Ltd, Japan) and commercial diagnostic kits (Beijing North Biotechnology Research Institute, Beijing, China). Levels of SOD and MDA, which can reflect the level of ROS, were detected by a RIA kit (Beijing Kangyuan Ruide Biotechnology Co. Ltd., Beijing, China) following the instructions of the company.

### Measurement of NT-proBNP by enzyme-linked immunosorbent assay (ELISA)

Level of NT-proBNP in serum was measured using ELISA kit (Cloud-Clone Corp, USA.) in Beijing Kangyuan Ruide Biotechnology Co. Ltd., (Beijing, China) according to the manufacturer’s instruction. Standards at a series of concentrations were run in parallel with the samples and the concentrations in the samples were calculated in reference to the corresponding standard curves. Six samples in each group were assayed in duplicate.

### Terminal Deoxynucleotidyl Transferased UTP Nick End Labeling (TUNEL)

To measure apoptotic activity of cardiomyocytes, heart tissues were incubated with TUNEL reaction mixture (Roche, Germany) at 37 °C for 1 hour. This was immediately followed by incubation with primary antibody of α-actin (1:500, Sigma) overnight at 4 °C. The nuclei were counter stained by DAPI in the mounting media. Laser scanning confocal microscopy (Olympus) was used to detect TUNEL-positive cells in randomly selected fields.

### The chemical analysis of QSKL by HPLC

Freeze-dried powder of QSKL was weighed accurately (0.04 g) and placed into a 1.5 ml centrifuge tube containing 1.0 ml 50% aqueous methanol for 5 minutes in a vortex. Following centrifugation at 12000 rpm for 10 minutes in a centrifuge (Eppendorf, Melbourne, Australia), supernatant (200 µl) was 5-fold diluted with 50% aqueous methanol and centrifuged at 12000 rpm for 10 minutes. The HPLC analysis was carried out on a Shimadzu HPLC (two LC-20ADXR solvent delivery units, a SIL-20ACXR auto-sampler, a CTO-20AC column oven, a SPD-M20A PDA detector, a DGU-20A3R degasser, and a CBM-20A controller). The chromatographic separation was performed on a Shiseido C18 column (150 × 2.1 mm, 2.7 μm) at 35 °C. 0.02% aqueous formic acid (A) and acetonitrile containing 0.02% formic acid (B) were used as the mobile phase for analysis. The flow rate was set at 0.4 ml/min. The elution condition was applied with a gradient program as follows: 0–20 min, 2–18% B; 20–30 min, 18–30% B; 30–35 min, 30–45% B; 35–40 min, 45–65% B; 40–55 min, 65–95% B; 55–60 min, 95% B. 10 μl were injected into HPLC system for analysis (Supplement 3).

### Cell culture and establishment of oxygen-glucose deprivation/recovery (OGD/R) injury model

H9C2 cells line was obtained from China Infrastructure of Cell Line Resources (Beijing, China). The H9C2 cells were maintained in high glucose DMEM supplemented with 10% FBS, 100 U/ml penicillin and 100 μg/ml streptomycin in a humidified incubator with 5% CO_2_ at 37 °C. Cells were divided into several groups: the control group, OGD/R-induced model group and QSKL-treated group. When H9C2 cells were cultured to 70–80% confluence, cells in model group were washed twice with PBS and earle’s balanced salt solution was added to imitate glucose deprivation. Then the plate was placed in an incubator, in which oxygen was deprived. After 8 hours, the plate was taken out, earle’s balanced salt solution was removed and DMEM with 10% FBS was added to recover oxygen-glucose for 12 hours. Cells in QSKL group were treated in the similar way, except that the culture media contained QSKL (400, 600, 800 μg/ml). In control group, fresh nutrient solution was changed at the time points of 8 and 12 hours.

### Cell viability analysis

H9C2 cells were seeded in 96-well plates at a density of 8 × 10^3^ cells per well. When cells were cultured to 70–80% confluence, the model of OGD/R was induced. After that, cells viability was determined by MTT assay. Cells were cultured with MTT solution dissolved in DMEM (0.5 mg/ml). Four hours later, the supernatants were removed and 150 μl DMSO was added to each well to dissolve the precipitate. Then the absorbance of each well was measured with a microplate reader (Thermo, USA) at a wavelength of 492 nm.

### Analysis of reactive oxygen species (ROS)

H9C2 cells were seeded at a density of 8 × 10^4^ cells/ml in 6-well plates and cellular production of ROS was measured by DCFH-DA assay, an oxidant-sensitive probe. After different incubations, H9C2 cells were washed three times with PBS, followed by incubation with DCFH-DA (10 μM) at 37 °C for half an hour. Then fluorescent intensity was detected under a fluorescence microscope (OLYMPUS, Japan). Image J was used to analyze the data.

### Determination of cells apoptosis by Hoechst 33342 assay

Hoechst staining method was applied to evaluate cells apoptosis. Cells were divided into five groups: the control group, the model group, the QSKL group, the Tiron group and the LY294002 group. The dosages of Tiron and LY294002 were 10 mM and 20 μM respectively according to the previous studies^33,34^. Treatments of cells in the control group, the model group and the QSKL group were similar as described above. Cells in the Tiron group were treated with Tiron for one hour before undergoing OGD/R process. Cells in LY294002 group were treated with LY294002 for one hour and other incubation conditions were the same as in the control group. After different treatments, H9C2 cells were washed three times with PBS, followed by incubation with Hoechst 33342 (10 μg/ml) at 37 °C for 15 minutes according to the manual. Stained cells were imaged under a fluorescent microscope (OLYMPUS, Japan). Six photographs were selected randomly, and the numbers of apoptotic cells were counted.

### Measurement of mitochondrial membrane potential (ΔΨm) levels

The reduction of mitochondrial membrane potential is an early manifestation of apoptosis. Mitochondria of H9C2 cells were stained with JC-1 (5,5′,6,6′-Tetrachloro-1,1′,3,3′-tetraethyl-imidacarbocyanine iodide). Cells were incubated with JC-1 dissolved in DMEM at 37 °C for 20 minutes, then washed twice with iced JC-1 staining buffer solution. The fluorescence was measured by a confocal laser scanning microscope (OLYMPUS, Japan). When there is normal mitochondrial membrane potential, JC-1 will diffuse into mitochondria and form polymers, emitting red fluorescence. When the mitochondrial membrane potential is reduced, JC-1 will exist in the cytoplasm in the form of monomer, emitting green fluorescence. The fluorescence was measured by a confocal laser scanning microscope (OLYMPUS, Japan). The ratio of red/green was calculated to indicate the loss of mitochondrial membrane potential.

### Effect of LY294002 on cell viability

Cells were divided into five groups: the control group, the model group, the QSKL group, the QSKL + LY294002 group and the LY294002 group. The procedure in the former three groups was similar as described above. Cells in QSKL + LY294002 group were treated in the similar way as those in QSKL group, except that the culture media contained LY294002 one hour before undergoing OGD/R process. Cells in LY294002 group were treated in the similar way as those in control group, except that the culture media contained LY294002. After different incubations, MTT assay was taken to measure cell viability.

### Western Blotting analysis on Akt pathway and apoptosis-related proteins

Apoptosis-related proteins were detected both by *in vivo* and *in vitro* studies. To evaluate the effect of QSKL *in vivo*, rats were divided into three groups including sham group, model group, and QSKL group. To investigate the mechanisms of QSKL on Akt pathway *in vitro*, cells were divided into four groups including control group, model group, QSKL group and QSKL + LY294002 group. Cells in the control group were treated with standard conditions, whereas cells in other groups were treated by OGD/R process. The collected myocardial tissues or cells were prepared with RIPA buffer (PPLYGEN, China) and proteins were extracted according to the manufacture’s instruction. Protein contents were measured with BCA protein assay kit (PPLYGEN, China). After addition of loading buffer and boiled for 5 minutes, samples were separated by 10% SDS-PAGE and transferred to NC membranes (Millipore, Germany). After being blocked with 5% non-fat dry milk for 2 hours, the membranes were incubated with different primary antibodies overnight at 4 °C. After washing with TBST three times, the membranes were incubated with HRP-conjugated secondary antibodies for 1 hour at room temperature. Washed three times with TBST, the proteins were detected with an enhanced chemiluminescence agent (GE, USA) and quantified by densitometry using an image analyzer (Bio-Rad, USA). Mouse anti-β-actin monoclonal antibody and mouse anti-GAPDH monoclonal antibody were served as an internal control.

The primary antibodies included rabbit monoclonal antibodies against cleaved-caspase-3 (1:1000), p-PTEN (1:1000), p-PI3K (1:1000) and Bax (1:1000), rabbit polyclonal antibody against Bcl-2 (1:1000), Akt (1:500), PTEN (1:1000) and PI3K (1:1000); mouse monoclonal antibodies against p-Akt (Ser473, 1:1000), p53 (1:1000) and secondary antibodies (goat anti-rabbit, 1:5000; goat anti-mouse, 1:5000).

### Statistical analysis

Data were expressed as the mean ± standard deviation (SD). Statistical analysis was undertaken by one way analysis of variance (ANOVA) and Dunnett’s test. Differences between groups were considered as statistically significant when *P* < 0.05.

## Electronic supplementary material

Supplement 1

Supplement 2

Supplement 3

Supplement 4
