# Supplementary material for: QSKL protects against myocardial apoptosis on heart failure via PI3K/Akt-p53 signaling pathway
Source: Sci Rep. 2017 Dec 5;7:16986. doi: 10.1038/s41598-017-17163-x (PMC5717266; doi:10.1038/s41598-017-17163-x)
Supplement: Supplementary file 2 — Supplement 2 [file 41598_2017_17163_MOESM2_ESM.docx]

**QSKL protects against myocardial apoptosis on heart failure via PI3K/Akt-p53 signaling pathway**

Hong Chang^1, 2,†^, Chun Li^3,†^,Qiyan Wang^1†^, Linghui Lu^1^,Qian Zhang^1^, Yi Zhang^4^, Na Zhang^3^, Yong Wang^1,*^, Wei Wang^5,*^

^1^ School of Life Sciences, Beijing University of Chinese Medicine, Bei San Huan Dong Lu 11, ChaoYang District, Beijing 100029, China

^2^Traditional Chinese Medicine College, North China University of Science and Technology, No. 21 Bohai Road, Caofeidian New City, Tangshan, Hebei, 063210, China

^3^ Modern Research Center for Traditional Chinese Medicine, Beijing University of Chinese Medicine, Bei San Huan Dong Lu 11, ChaoYang District, Beijing 100029, China

^4^ School of Chinese Materia Medica, Beijing University of Chinese Medicine, Bei San Huan Dong Lu 11, ChaoYang District, Beijing 100029, China

^5^ Beijing University of Chinese Medicine, Bei San Huan Dong Lu 11, ChaoYang District, Beijing 100029, China

^* Corresponding authors: Wei Wang, Tel.: +86 10 6428 6508; E-mail: wangwei26960@126.com (W. Wang)^

^Yong Wang, Tel.: +86 10 6428 6180; E-mail: doctor_wangyong@163.com (Y. Wang)^

**^†^**^: These authors contributed to the paper equally.^

We applied network pharmacology to evaluate the effective ingredients and respective targets of QSKL, and selected ingredients related to apoptosis. The results showed that six ingredients, including quercetin, kaempferol, luteolin, β-sitosterol, tanshinone IIA and naringenin, have the potential anti-apoptotic effect. The detailed methods and results are shown as below for your reference.

**Pharmacology Methods:**

TcmSP^[1]^, Traditional Chinese Medicines for Systems Pharmacology Database and Analysis Platform based on pharmacokinetics (absorption, distribution, metabolism and excretion, ADME) was applied in the experiment. Six herbal medicines of QSKL, including Radix Astragali Mongolici, Salvia miltiorrhiza bunge, Flos Lonicerae, Scrophularia, Radix Aconiti Lateralis Preparata and Radix Glycyrrhizae, were entered into TcmSP and all the ingredients of QSKL and targets related to ingredients were extracted from the database^[2-3]^. Based on oral bioavailability (OB)≥30% and drug likeness (DL) ≥0.2, another selection was applied. Among the targets related to apoptosis, we found the corresponding active ingredients.

**Results:**

We gained 572 bioactive ingredients and 656 protein targets closely related to cardiovascular disease. According to the OB value ≥30% and DL value ≥0.2, 173 bioactive ingredients and 283 protein targets were chosen(Table 1). The former six bioactive ingredients of which corresponded to most protein targets were quercetin, kaempferol, luteolin, β-sitosterol, tanshinone IIA and naringenin(Table 2). According to the targets related to apoptosis, including caspase-3, Bcl-2, Bax, and p53, we found the corresponding active ingredients(Table 3). In the future studies, we will validate the effects of these potentially effective ingredients by in vivo and in vitro experiments.

Table 1 Number of drug ingredients

| alternative condition  Herbal medicine | Number of ingredients | | |
| --- | --- | --- | --- |
|  | The whole formula | | OB≥30%，DL≥0.2 |
| Salvia miltiorrhiza bunge | 167 | | 61 |
| Radix Aconiti Lateralis Preparata | | 21 | 8 |
| Radix Glycyrrhizae | 169 | | 82 |
| Radix Astragali Mongolici | 62 | | 17 |
| Flos Lonicerae | 175 | | 14 |
| Scrophularia | 38 | | 5 |

Table 2 The ingredients and number of corresponding protein targets

| Ingredients | Number of targets |
| --- | --- |
| Quercetin | 166 |
| Kaempferol | 74 |
| Luteolin | 68 |
| beta-sitosterol | 57 |
| Tanshinone IIA | 53 |
| Naringenin | 48 |

Table 3 Distribution of corresponding ingredients and protein targets

| Protein targets | Corresponding ingredients |
| --- | --- |
| Caspase-3 | luteolin |
|  | tanshinone IIA |
|  | kaempferol |
|  | naringenin |
|  | quercetin |
|  | beta-sitosterol |
| Bcl-2 | luteolin |
|  | quercetin |
| Bax | kaempferol |
|  | quercetin |
|  | beta-sitosterol |
| P53 | luteolin |
|  | tanshinone IIA |
|  | quercetin |

References:

[1]RU J, LI P, WANG J, et al. TCMSP: a database of systems pharmacology for drug discovery from herbal medicines [J]. Journal of cheminformatics, 2014, 6(13.

[2]LI B, XU X, WANG X, et al. A systems biology approach to understanding the mechanisms of action of chinese herbs for treatment of cardiovascular disease [J]. International journal of molecular sciences, 2012, 13(10): 13501-20.

[3]TAO W, XU X, WANG X, et al. Network pharmacology-based prediction of the active ingredients and potential targets of Chinese herbal Radix Curcumae formula for application to cardiovascular disease [J]. Journal of ethnopharmacology, 2013, 145(1): 1-10.
