# Supplementary material for: QSKL protects against myocardial apoptosis on heart failure via PI3K/Akt-p53 signaling pathway
Source: Sci Rep. 2017 Dec 5;7:16986. doi: 10.1038/s41598-017-17163-x (PMC5717266; doi:10.1038/s41598-017-17163-x)
Supplement: Supplementary file 3 — Supplement 3 [file 41598_2017_17163_MOESM3_ESM.docx]

**QSKL protects against myocardial apoptosis on heart failure via PI3K/Akt-p53 signaling pathway**

Hong Chang^1, 2,†^, Chun Li^3,†^,Qiyan Wang^1†^, Linghui Lu^1^,Qian Zhang^1^, Yi Zhang^4^, Na Zhang^3^, Yong Wang^1,*^, Wei Wang^5,*^

^1^ School of Life Sciences, Beijing University of Chinese Medicine, Bei San Huan Dong Lu 11, ChaoYang District, Beijing 100029, China

^2^Traditional Chinese Medicine College, North China University of Science and Technology, No. 21 Bohai Road, Caofeidian New City, Tangshan, Hebei, 063210, China

^3^ Modern Research Center for Traditional Chinese Medicine, Beijing University of Chinese Medicine, Bei San Huan Dong Lu 11, ChaoYang District, Beijing 100029, China

^4^ School of Chinese Materia Medica, Beijing University of Chinese Medicine, Bei San Huan Dong Lu 11, ChaoYang District, Beijing 100029, China

^5^ Beijing University of Chinese Medicine, Bei San Huan Dong Lu 11, ChaoYang District, Beijing 100029, China

^* Corresponding authors: Wei Wang, Tel.: +86 10 6428 6508; E-mail: wangwei26960@126.com (W. Wang)^

^Yong Wang, Tel.: +86 10 6428 6180; E-mail: doctor_wangyong@163.com (Y. Wang)^

**^†^**^: These authors contributed to the paper equally.^


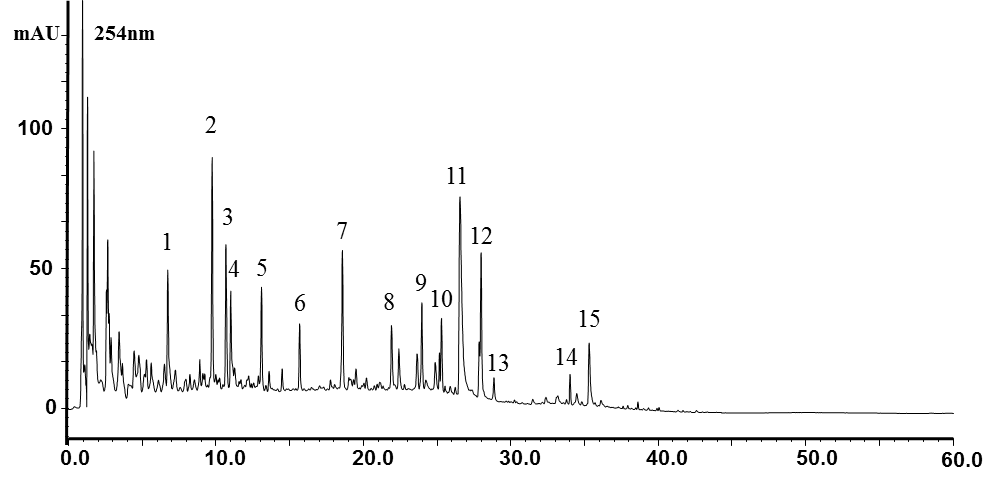


**Supplement 3 | Qualitative analysis on freeze-dried powder of QSKL.** HPLC-PDA chromatograms numbered from 1 to 15 represent chlorogenic acid, cryptochlorogenin acid, neochlorogenic acid, secologanic acid, sweroside, secoxyloganin, liquiritin, isochlorogenic acid A, isochlorogenic acid C, ononin, salvianolic acid B, calycosin, harpagoside, formononetin and glycyrrhizic acid.
