# Supplementary material for: QSKL protects against myocardial apoptosis on heart failure via PI3K/Akt-p53 signaling pathway
Source: Sci Rep. 2017 Dec 5;7:16986. doi: 10.1038/s41598-017-17163-x (PMC5717266; doi:10.1038/s41598-017-17163-x)

**Supplementary Figures**

**QSKL protects against myocardial apoptosis on heart failure via PI3K/Akt-p53 signaling pathway**

Hong Chang, Chun Li, Qiyan Wang, Linghui Lu, Qian Zhang, Yi Zhang, Na Zhang, Yong Wang, Wei Wang

**Supplementary Figure 1:** Full gel images for Figures 2


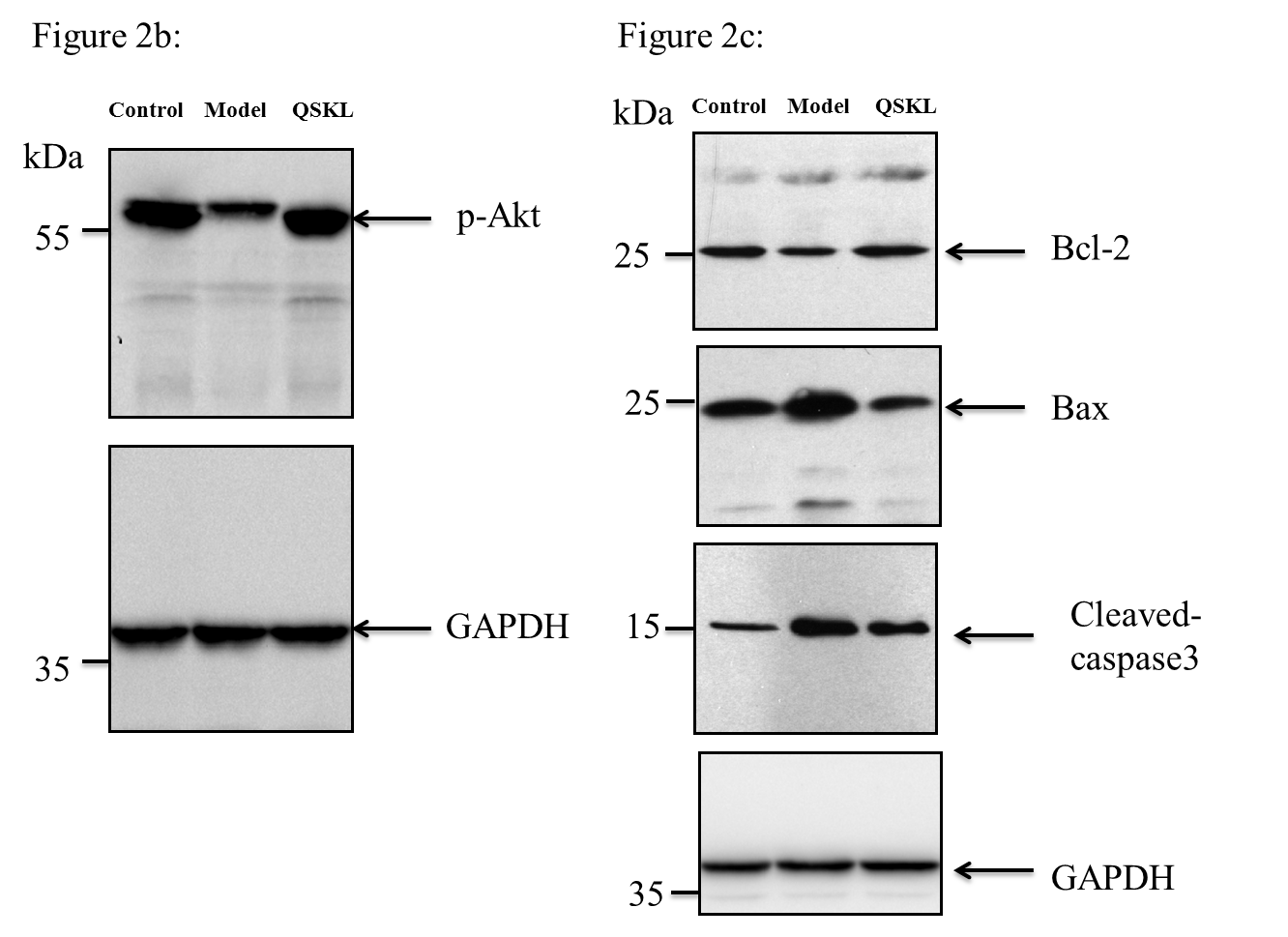


**Supplementary Figure 2:** Full gel images for Figures 7


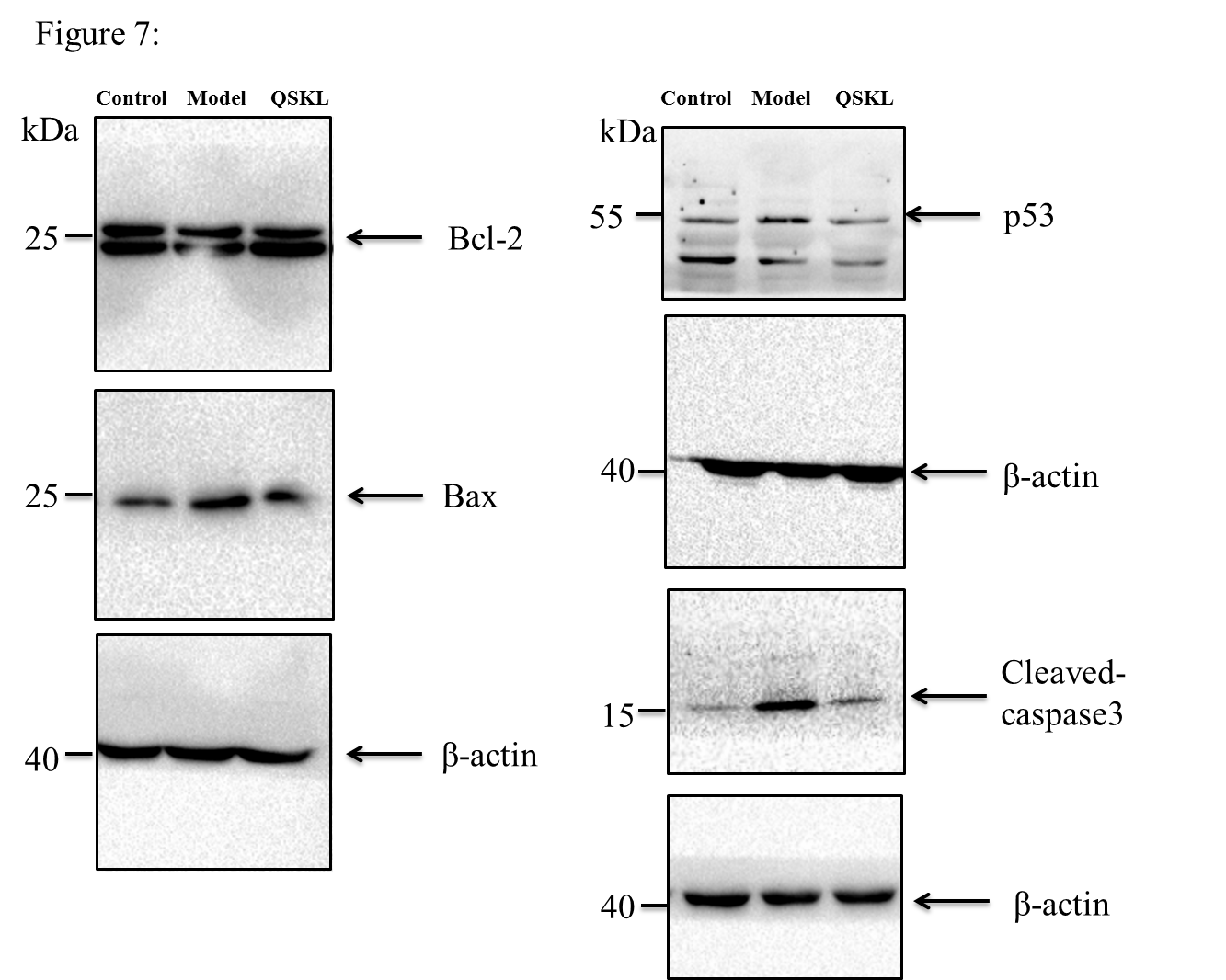


**Supplementary Figure 3:** Full gel images for Figures 8


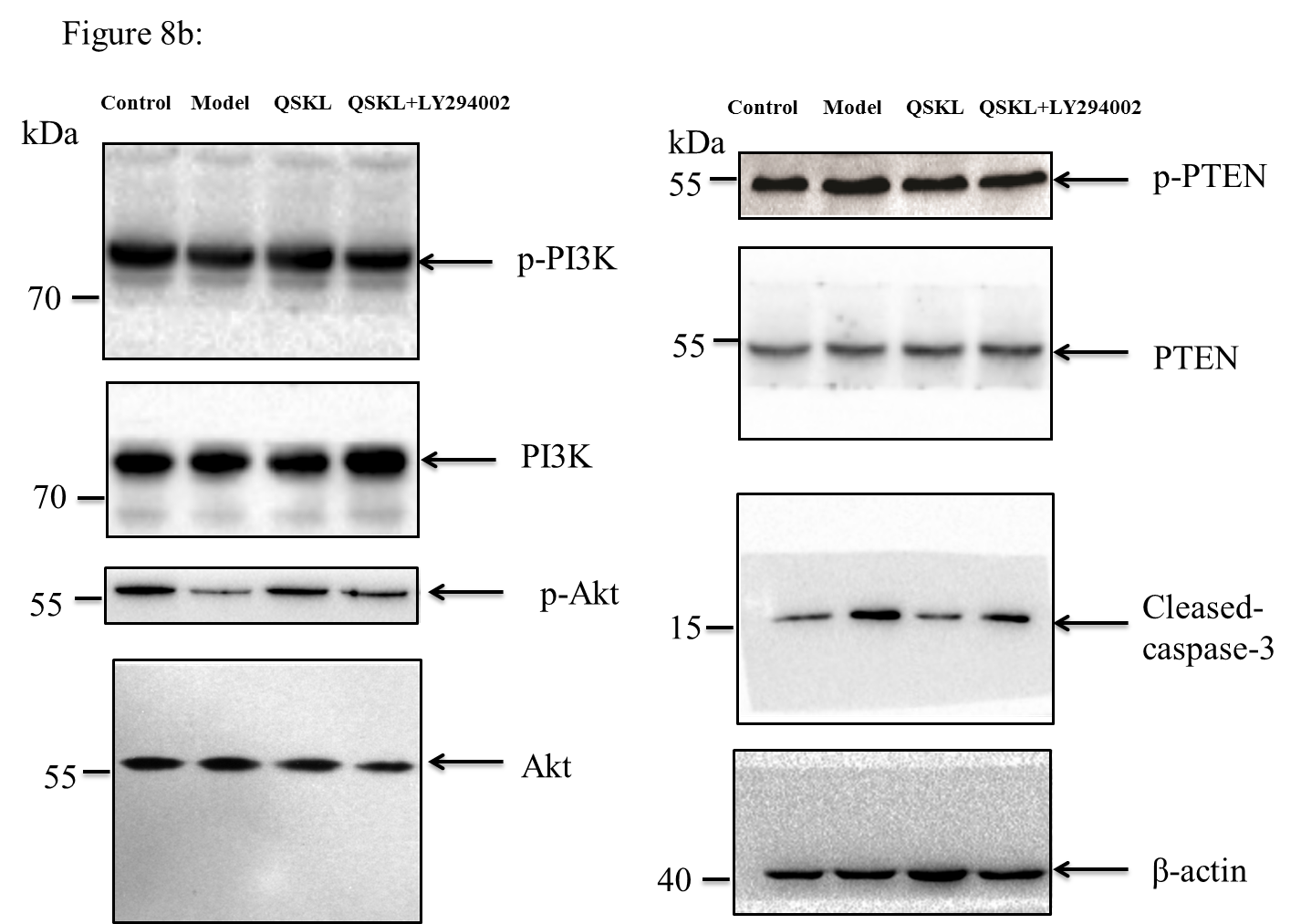

Supplement: Supplementary file 4 — Supplement 4 [file 41598_2017_17163_MOESM4_ESM.doc]
